# Brain pericytes from stress-susceptible pigs increase blood-brain barrier permeability *in vitro*

**DOI:** 10.1186/2045-8118-9-11

**Published:** 2012-06-29

**Authors:** Elodie Vandenhaute, Maxime Culot, Fabien Gosselet, Lucie Dehouck, Catherine Godfraind, Michel Franck, Jean Plouët, Roméo Cecchelli, Marie-Pierre Dehouck, Marie-Magdeleine Ruchoux

**Affiliations:** 1 Univ Lille Nord de France, F-59000, Lille, France; 2 UArtois, LBHE, F-62300, Lens, France; 3 Département de neuropathologie, Clinique Saint-Luc, ULB, 1200, Bruxelles, Belgium; 4 Ecole Nationale Vétérinaire de Lyon, Laboratoire de Zootechnie, F-69280, Marcy l’Etoile, France; 5 Inserm, U 689, Centre de Recherche Cardiovasculaire Inserm Lariboisière, 75475, Paris Cedex 10, France; 6 CEA, DSV/DRM, Groupe d'Innovation Diagnostique et Thérapeutique sur les Infections à Prions, 18 Route du Panorama, BP 6, 92265, Fontenay aux Roses Cedex, France; 7 Present address: Mannheim Medical Faculty, University of Heidelberg, Childrens Hospital, Theodor-Kutzer-Ufer 1-3, D-68167, Mannheim, Germany

**Keywords:** Blood–brain barrier, Endothelial permeability, Neurovascular unit, Pericytes, Perivascular spaces, Porcine stress syndrome

## Abstract

**Background:**

The function of pericytes remains questionable but with improved cultured technique and the use of genetically modified animals, it has become increasingly clear that pericytes are an integral part of blood–brain barrier (BBB) function, and the involvement of pericyte dysfunction in certain cerebrovascular diseases is now emerging. The porcine stress syndrome (PSS) is the only confirmed, homologous model of malignant hyperthermia (MH) in veterinary medicine. Affected animals can experience upon slaughter a range of symptoms, including skeletal muscle rigidity, metabolic acidosis, tachycardia and fever, similar to the human syndrome. Symptoms are due to an enhanced calcium release from intracellular stores. These conditions are associated with a point mutation in *ryr1/hal* gene, encoding the ryanodine receptor, a calcium channel. Important blood vessel wall muscle modifications have been described in PSS, but potential brain vessel changes have never been documented in this syndrome.

**Methods:**

In the present work, histological and ultrastructural analyses of brain capillaries from wild type and *ryr1* mutated pigs were conducted to investigate the potential impairment of pericytes, in this pathology. In addition, brain pericytes were isolated from the three porcine genotypes (wild-type NN pigs; Nn and nn pigs, bearing one or two (n) mutant *ryr1/hal* alleles, respectively), and tested *in vitro* for their influence on the permeability of BBB endothelial monolayers.

**Results:**

Enlarged perivascular spaces were observed in *ryr1*-mutant samples*,* corresponding to a partial or total detachment of the astrocytic endfeet. These spaces were electron lucent and sometimes filled with lipid deposits and swollen astrocytic feet. At the ultrastructural level, brain pericytes did not seem to be affected because they showed regular morphology and characteristics, so we aimed to check their ability to maintain BBB properties *in vitro*. Our results indicated that pericytes from the three genotypes of pigs had differing influences on the BBB. Unlike pericytes from NN pigs, pericytes from Nn and nn pigs were not able to maintain low BBB permeability.

**Conclusions:**

Electron microscopy observations demonstrated brain capillary modifications in PSS condition, but no change in pericyte morphology. Results from *in vitro* experiments suggest that brain pericytes from *ryr1* mutated pigs, even if they are not affected by this condition at the ultrastructural level, are not able to maintain BBB integrity in comparison with pericytes from wild-type animals.

## Background

The porcine stress syndrome (PSS) is linked to a single point mutation in the skeletal muscle *ryr* gene, which leads to the replacement of a cytosine by a thymine molecule at nucleotide 1843 [1]. Upon slaughter, pigs carrying the halothane sensitivity (n) allele at the *ryr1* locus can exhibit rapid-onset, severe malignant hyperthermia (MH), experiencing a range of symptoms, including skeletal muscle rigidity, metabolic acidosis, tachycardia and fever, which are due to an enhanced calcium release from intracellular stores. This condition has been studied because these animals typically produce pale, soft and exsudative pork, which is responsible for a poor meat quality and has huge consequences in meat processing industry. In 2007, Franck *et al.*[2] demonstrated that pigs carrying one or two mutant (n) alleles at the *ryr1* locus exhibited blood vessel wall changes, in comparison with wild-type NN swine. In skeletal muscle vessels, the media was particularly disorganized. As *ryr*1 is expressed throughout the brain, central nervous system involvement in PSS has been suggested [3,4]. In view of the vascular aspect of PSS in skeletal muscle vessels [2], we decided to investigate potential brain capillary changes in stress-susceptible pigs. Brain capillaries are the anatomical basis of the blood–brain barrier (BBB), which is the focal point of the neurovascular unit (NVU) comprising endothelial cells and pericytes at the capillary level, and also astrocytes, oligodendrocytes, microglia and neurons. Interactions between these different actors are essential for maintaining cerebral homeostasis. Any dysfunction of a component of the NVU is associated with an alteration of cellular interactions within the NVU. This dysfunction is associated with neurologic diseases such as stroke and has also been suggested in Alzheimer’s disease [5].

To study BBB in PSS brains, ultrastructural investigations of the cerebral capillaries were performed in the three *ryr1* genotypes of pigs (NN, wild type; Nn and nn, carrying one and two mutant alleles respectively). Cellular and molecular interactions between different cell types within the BBB can more easily be studied *in vitro*. However, there has been a delayed interest in pericytes and the major reason is due to the fact that to obtain pure endothelial cell cultures, pericytes have to be eliminated during the isolation process. Now that cell culture techniques allow the production of pure endothelial cultures on the one hand and of pure pericyte cultures on the other hand, pericytes can easily be studied *in vitro*, alone or in coculture with other cell types [6-8]. *In vitro* models using both brain endothelial cells and brain pericytes have shown that pericytes are able to regulate tight junction (TJ) protein expression in endothelial cells (ECs) [9-11], and also the expression of transporters [12,13]. Therefore in this study, brain pericytes were extracted from the three porcine genotypes and studied for their influence on BBB permeability in an *in vitro* BBB model [14], in which the various cell types can be assembled.

## Methods

### Animals

Twenty-one pigs from 100 to 120 kg were used in this study. They were provided by FranceHybride (Orléans, France), were kept in animal house in the National Veterinary School in Lyon (France) and handled in accordance with national guidelines. Pigs (females and castrated males) were undertaken from crosses between the LargeWhite Pietrain and LargeWhite Landrace populations. Genotypes of all pigs were previously determined with a DNA test by PCR RSLP method in Labogena laboratory (INRA, Jouy-en-Josas, France). Three groups were studied: a group of 7 NN (homozygous-halothane-negative or stress resistant), a group of 7 Nn (heterozygous halothane-negative or stress carrier) and a group of 7 nn (halothane positive or stress-susceptible) respectively to the *hal/ryr1* locus. All of them were Rn ++ at the Rn locus. From 100 to 120 kg live weight, these pigs were slaughtered after general anaesthesia (Zoletil ® 100, tiletamin 8 mg/kg in IM, myorelaxation with Nesdonal ® IG, Thiopental sodic 20 mg/lg in IV).

### Electron microscopy investigations

Brain fragments (2 mm length × 1 mm diameter from the frontal lobe, anterior part of the frontal 1 and from the cerebellum, lateral part of the superior semilunar lobule) were fixed in CARSON liquid [15]. Then they were transferred in 2% osmium tetroxide in 0.1 M phosphate buffer, later dehydrated in acetone and embedded in Epon 812. Finally they were sectioned and stained using standard protocols with uranyl acetate and lead citrate. Brain capillaries were observed morphometrically in randomly selected fields in each of the above-mentioned brain regions from the animals of each group (NN, Nn and nn).

### Cell culture

#### *Preparation of the* in vitro *BBB model*

The method from Dehouck *et al.*[14] and Cecchelli *et al.*[16] was used. Use of animals for *in vitro* experiments was done according to the French Veterinary council’s guide (approval n° B62-498-5).

Briefly, glial cells were isolated from newborn Sprague–Dawley rats (Centre d’Élevage Roger Janvier, Le Genest Saint Isle, France), plated on 6-well dishes (Ref: 3506, Costar, Corning Incorporated, NY, USA) and cultured during 3 weeks in DMEM (Dulbecco’s Modified Eagle’s Medium, Ref: 31600–083, Life Technology, Carlsbad, California, USA) supplemented with 10% Foetal Calf Serum (v/v) (Hyclone Laboratories, Logan, UT, USA), 2 mM L-glutamine (Merck Chemicals, Darmstadt, Germany) and 50 μg.mL^-1^ gentamicin (Ref: A2712, Biochrom AG, Berlin, Germany). Glial cells cultures were stabilised three weeks after seeding, and consisted of a mix of astrocytes, oligodendrocytes and microglial cells. The astrocytic population was characterized by GFAP (*Glial Fibrillary Acidic Protein*) expression: 60% of glial cells were GFAP positive as shown by immunostaining (data not shown). From this stage, they were used to set up the cocultures with endothelial cells.

Bovine brain capillary endothelial cells were isolated as previously described [14], and seeded onto rat tail collagen gel coated inserts (CM Inserts, diameter 30 mm, 0.4 μm pore size, Millicell-CM, Millipore Corporation, Molsheim, France) which were adapted in the wells containing glial cells. Experiments were initiated after 12 days of coculture between the two kinds of cells. This time course allowed the endothelial cells to acquire a proper BBB phenotype [14].

The culture medium corresponded to DMEM supplemented with 10% (v/v) Horse serum (Ref: 7399488D, InvitroGen Corporation, Paisley, Scotland, UK), 10% (v/v) Calf serum (Ref: 7316368D, InvitroGen Corporation), 2 mM L-glutamine, 50 μg/mL gentamicin and 1 ng/ml basic fibroblast growth factor (bFGF, Ref: F0291, Recombinant Human bFGF, Sigma-Aldrich, Saint Quentin Fallavier, France).

#### Isolation of porcine and bovine brain pericytes

For each pig genotype, the whole hemisphere of the brain was dissected. Meninges and white matter were removed and the grey matter was cut into 2-mm^3^ fragments and washed twice with phosphate-buffered saline solution (PBS). These fragments were resuspended in 2 volumes of PBS and homogenized by up and down strokes in a 40 mL-glass homogenizer fitted with a large clearance glass pestle (0.152 mm clearance). The resulting homogenate was passed through a nylon sieve (180-μm pore size) and the filtrate - containing the microvessels - was homogenized with a second glass pestle with a smaller clearance (0.076 mm clearance). Finally, the microvessels were collected on a new nylon sieve (60-μm pore size) and washed abundantly with PBS.

Microvessels were then removed from the 60-μm-pore mesh and resuspended in buffer (Hank’s buffer supplemented with 10 mM Hepes and 0.1% (w/v) bovine serum albumin). After centrifugation (1000 g, 7 min at room temperature) microvessels were digested with a mix collagenase dispase (Roche Diagnostics, Meylan, France) / Dnase I (Roche Diagnostics) / TLCK (Tosyl-L-Lysine Chloromethyl Ketone, Sigma-Aldrich) for 30 minutes at 37°C under agitation. Digested fragments of microvessels were washed and then seeded onto 60-mm-diameter dishes coated with Matrigel (Matrigel growth factor reduced, Becton Dickinson; dilution 1/50 in DMEM). Primary cultures were rapidly overgrown by pericytes. Pericytes were subcultured at a split ratio 1/10 and used at passages ≤ 3. The growth medium for pericytes was DMEM supplemented with 20% (v/v) Foetal Calf Serum (Hyclone Laboratories, Logan, UT, USA), 2 mM L-glutamine, 50 μg/mL gentamicin and 1 ng/ml bFGF.

#### Culture of pericytes

A mix of endothelial cells and pericytes were grown from digested microvessels. Culture medium was refreshed twice a week. After 5 days of culture, cells were passaged with trypsin (0.05%)/EDTA (EthyleneDiamineTetraAcetic acid, 0.02%) (L2143, Biochrom AG) and subcultured. As pericytes are more resistant and grow faster than endothelial cells, hardly any endothelial cells subsist after the first passage. After 2 days of culture, pericytes have completely overgrown endothelial cells. Pericytes are frozen in freeze medium containing DMEM supplemented with 30% (v/v) Foetal Calf Serum (Hyclone Laboratories, Logan, UT, USA), 2 mM L-glutamine, 50 μg/mL gentamicin and 10% (v/v) DMSO.

For experiments, 50,000 pericytes of each porcine genotype (NN, Nn, and nn) were seeded on gelatin-coated 6-wells plates (Nunc, Roskilde, Denmark). Healthy bovine pericytes were also extracted according to the same protocol, and were used for experiments to confirm the absence of species-related variations concerning the response. The resulting cultures of porcine and bovine pericytes were glial fibrillary acidic protein (GFAP)-negative (data not shown) and α-smooth muscle actin (α-SMA)-positive, as assessed by immunostaining.

#### In vitro *model for interaction between pericytes and endothelial cellsc*

After 12 days of coculture with glial cells, the differentiated endothelial cell monolayers were separated from glial cells and transferred onto 6-well plates with pericytes. For each genotype of pericytes (NN, Nn and nn porcine pericytes, and bovine pericytes), a triplicate of cocultures was prepared. The two cell types were left to interact *via* soluble factors over a 4-day period in DMEM supplemented with 10% (v/v) Horse serum, 10% (v/v) Calf serum, 2 mM L-glutamin, 50 μg/mL gentamicin and 1 ng/ml bFGF.

### Immunofluorescence studies on pericyte cultures

The three genotypes of pericytes were double-immunostained for α-SMA and nerve-glial antigen 2 (NG2), two markers used to identify pericytes in culture. The procedure was the following: cells were washed with PBS-CMF solution (phosphate buffered saline - calcium and magnesium free; 8.0 g.L^-1^ NaCl, 0.2 g.L^-1^ KCl, 0.2 g.L^-1^ KH_2_PO_4_ and 2.87 g.L^-1^ Na_2_HPO_4_(12H_2_O), pH 7.4) once, fixed in methanol/acetone (50%/50% v/v) for 1 min and then were washed three times with PBS-CMF. Following a 30-min incubation in PBS-CMF supplemented with 10% (v/v) normal goat serum (NGS, G6767, Sigma-Aldrich), cells were incubated with the first primary antibody for 1 h at room temperature (mouse anti-α-SMA, clone 1A4, Dako, Glostrup, Denmark; dilution 1/200 in PBS-CMF supplemented with 2% NGS. After this first incubation, preparations were washed three times in PBS-CMF supplemented with 2% NGS, and then incubated with the second primary antibody (rabbit anti-NG2, Millipore, Temecula, California, USA; dilution 1/200 in PBS-CMF supplemented with 2% NGS) for 1 h. After 3 washes in PBS-CMF supplemented with 2% NGS, preparations were consecutively incubated with the secondary antibodies for 1 h in the dark each (Alexa Fluor® 488-conjugated goat anti-rabbit IgG and Alexa Fluor® 568-conjugated anti-mouse IgG, Molecular Probes, Eugene, Oregon, USA; dilution 1/200 in PBS-CMF supplemented with 2% NGS). Nuclei were stained using Hoechst reagent (Hoechst 33258, ICN Pharmaceuticals) after secondary antibody labelling. Preparations were finally mounted with Mowiol (Sigma-Aldrich) supplemented with an anti-fading agent (Dabco, Ref: D2522, Sigma-Aldrich) and the staining was analyzed using a fluorescence microscope (Leica DMRD, Leica Microsystems, Wetzlar, Germany). Images were collected using a Cool SNAP RS Photometrics camera (Leica Microsystems). Images were processed and mounted using Adobe Photoshop software version 5.5 (Adobe Systems, San Jose, CA, USA).

### Trans-endothelial transport studies

The inserts (containing the endothelial monolayer, or only coated with collagen gel) were transferred into six-well plates containing 2.5 ml of Ringer-HEPES solution (150 mM NaCl, 5.2 mM KCl, 2.2 mM CaCl_2_, 0.2 mM MgCl_2_-6H_2_O, 6 mM NaHCO_3_, 5 mM HEPES, 2.8 mM glucose, pH 7.4) per well (abluminal compartment). The cell culture medium in the filter was removed, and 1.5 ml of Ringer-HEPES solution containing 50 μM lucifer yellow (LY, lucifer yellow CH dilithium salt, MW: 457, Ref L0259, Sigma-Aldrich) was added to the upper luminal compartment. Incubations were all performed at 37°C on a rotating platform. After 20, 40 and 60 min, the inserts were transferred into new wells to minimise the possible passage from the lower to the upper compartment. For each time point, a 200 μL aliquot from each lower compartment and a 20 μL aliquot from the stock solution of LY were placed in a fluorometer for quantification (Excitation wavelength: 425 nm ; Emission wavelength: 538 nm, Fluoroscan Ascent FL, Thermo Labsystems, Issy-Les-Moulineaux, France). The endothelial permeability coefficient (P_e_) of LY was calculated in centimetres/minute (cm/min), as described Siflinger-Birnboim *et al.*[17]. To obtain a concentration-independent transport parameter, the clearance principle is used. Briefly, the average volume cleared is plotted versus time, and the slope is estimated by linear regression. Both insert permeability (PS_f_, for insert only coated with collagen) and insert plus endothelial cell permeability (PS_t_, for insert with collagen and cells) were taken into consideration, according to the following formula:

(1) $$1/PS_{e}=1/PS_{t}-1/PS_{f}$$

The permeability value for the endothelial monolayer was then divided by the surface area of the porous membrane of the insert (Millicell CM, 0.4 μm pore size, membrane surface 4.2 cm^2^, Millipore Corporation, Carrigtwahill, Cork, Ireland) to obtain the endothelial permeability coefficient (P_e_) of the molecule (in cm.min^-1^). The reference was considered to be the endothelial – glial cells coculture; permeability coefficients obtained in other conditions were expressed in % according to this value.

LY transport allows endothelial monolayer integrity assessment after 16 days of co-culture with glial cells, or after a 12-day coculture with glial cells plus a 4-day coculture period with the different genotypes of pericytes.

### Determination of vascular endothelial growth factor levels in the culture supernatant

Because vascular endothelial growth factor (VEGF) is known to affect endothelial cell permeability, it was assayed in the coculture medium. After the final 48-h in the coculture period, the medium was collected and frozen at – 80°C. Medium in the filter (luminal compartment) and medium in the wells (abluminal compartment) were collected separately. VEGF concentrations (expressed as ng/ml) in the culture medium were determined by radio receptor assay as already described [18]. The results were expressed by comparison with the standard curve of human recombinant VEGF 165 amino acids diluted in the culture medium. Values are expressed as means ± SEM of VEGF concentration per mL of conditioned medium.

### Statistical analysis

All results are expressed as means ± SEM or ± SD from three or more independent experiments. Statistical significance was assessed by one-way ANOVA followed by Tukey test. A P-value < 0.05 was considered as significant. All statistical analyses were performed using GraphPad Prism version 5.0 for Windows (GraphPad Software, San Diego, California, USA).

## Results

### Ultrastructural findings in brain capillaries

**Figure 1:**
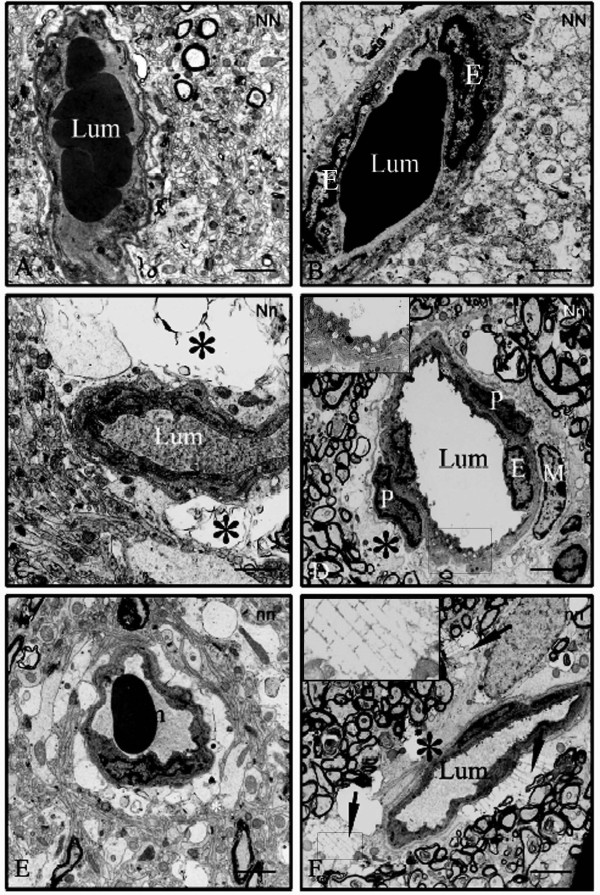
**Electron microscopy of cross sections of brain capillaries from pigs with or without a point mutation in the*ryr1/hal*gene: NN (A and B), Nn (C and D) and nn (E and F) pigs.** A and B: Cross sections of capillary segments from two NN pigs. The lumen (**Lum**) was surrounded by an extremely thin endothelium. There is no obvious large perivascular space (Original magnification: × 6000). C and D: Cross sections of capillary segments from two Nn pigs. The lumen (**Lum**) was surrounded by a thin endothelium (**E**), with some small intraluminal expansions and microvesicles (particularly in D). Inset: Higher magnification of the microvesicles. Note the enlarged perivascular spaces (*) in C and D, and the macrophage (**M**) close to the pericyte (**P**). (Original magnification: × 6000). E and F: Cross sections of capillary segments from two nn pigs. Oedematous changes in the pericapillary parenchyma can be seen in E. Pseudocrystalloid deposits (arrow) are visible in the perivascular space (*), between the capillary and the myelinated fascicles in F. Inset: Higher magnification of the pseudocristalloid. (Original magnification: ×6000). Scale bars = 4 μm for all micrographs.

The capillaries in control (NN) pigs presented a thin endothelium and a regular, luminal front (Figure 1A, 1B). No large perivascular spaces (PVSs) were noticed around the brain capillaries in NN pigs (Figure 1A, 1B). In contrast, Nn and nn brain capillaries always showed vessel wall structural changes (Figure 1C, 1D, 1E, 1F). Some intracytoplasmic vesicles were found (Figure 1D). The most striking changes concerned the PVSs (*, Figure 1C, 1F), which were large and filled with electron lucent vacuoles and/or swollen astrocytic endfeet (leading to the dissociation of the nearby parenchyma is some cases) (Figure 1C, 1F). In the Nn group, the lumen was surrounded by a thin endothelium (Figure 1C) exhibiting intraluminal expansion and intracytoplasmic vesicles (Figure 1D, inset), and macrophages (M) containing clear vacuoles were observed around the capillaries (Figure 1D); these cells were unambiguously identified by the absence of a defined basal lamina and the presence of numerous cytoplasmic lipid inclusions. In the nn group, oedematous changes were observed in the pericapillary parenchyma (Figure 1E) and electron lucent, pseudocrystalloid formations were observed close to the vessel wall or in the nearby parenchyma (arrows and inset, Figure 1F). These crystalloids probably correspond to an accumulation of lipid degradation products. However, the brain pericytes did not exhibit any noticeable modification at this level.

Since examination of the Nn and nn brains always revealed a wider PVS around capillaries (associated with signs of focal accumulation of lipids and slight vessel wall changes, relative to NN brains), we hypothesized that BBB changes could be involved in PSS. Indeed, the BBB is responsible for the maintenance of brain homeostasis at the capillary level. Brain capillaries in mutated pigs exhibited swollen astrocytic endfeet, reminiscent of an astrocyte modification in PSS, but pericytes did not show any major changes at the ultrastructural level. To investigate if physiological changes affect brain pericytes, we therefore aimed at checking their ability to maintain BBB properties. To investigate their functionality and influence on BBB endothelial cells, we used a well-characterized *in vitro* BBB model developed in our laboratory [14] to investigate permeability changes with brain pericytes extracted from the three genotypes of pigs.

### BBB permeability investigations

#### Identification and characterization of pericytes in vitro

**Figure 2:**
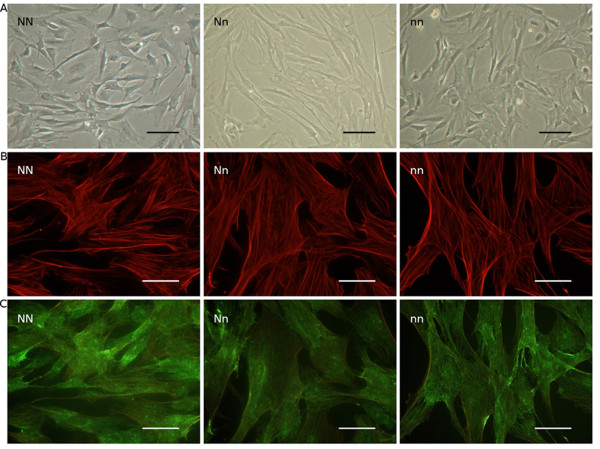
**Pericyte culture.** A: A phase-contrast micrograph of porcine brain microvascular pericytes from the different genotypes (NN, Nn and nn). Scale bar = 100 μm. B- Fluorescence micrograph of the same pericytes stained for alpha-smooth muscle actin. Scale bar = 50 μm. C- Fluorescence micrograph of brain porcine pericytes after nerve-glial antigen 2 (NG2) immunostaining. The three genotypes exhibited the same morphological and immunocytochemical features. Scale bar = 50 μm.

Brain microvascular pericytes were isolated from the three genotypes of pigs (NN, Nn and nn). Whatever the genotype studied, *in vitro*-cultured brain microvascular pericytes exhibited their characteristic irregular morphology (Figure 2A) and were positive for the alpha-smooth muscle actin (Figure 2B) and nerve-glial antigen 2 (NG2) (Figure 2C), which are markers previously used to identify this cell type [19,20]. Pericyte cultures were found negative for von Willebrand factor (vWF) and GFAP, demonstrating the absence of endothelial and glial contamination, respectively (data not shown). Pericytes were also characterized according to their gamma-glutamyl transpeptidase expression, as already described [21]. There were no differences between the three genotypes of pericytes in terms of either morphology or alpha-smooth muscle actin/NG2 staining.

Bovine and porcine pericytes from the three genotypes were also checked for *ryr*1 expression by real-time and semi-quantitative RT-PCR, but no amplification of *ryr*1 mRNA was found for pericytes, in contrast to skeletal muscle positive control cells (data not shown).

#### The BBB permeability assay

**Figure 3:**
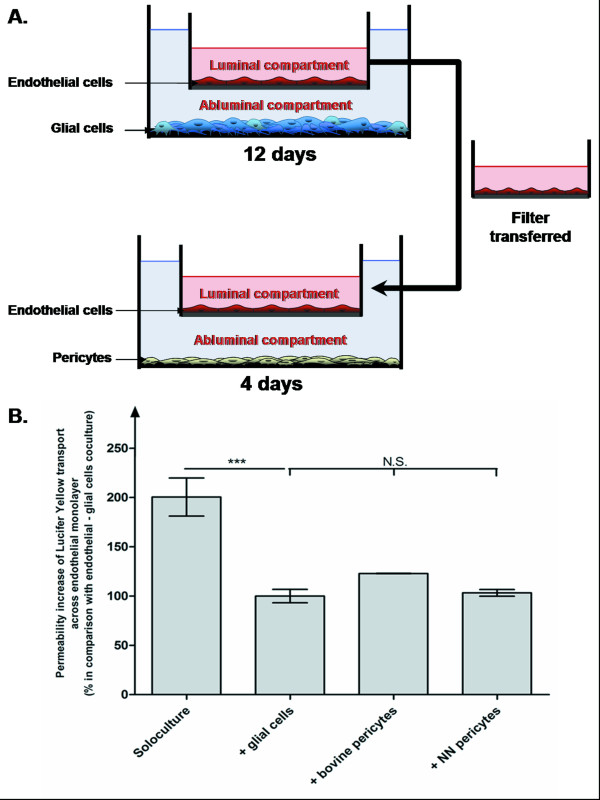
**The blood–brain barrier model used to test the effect of pericytes on BBB permeability.** A- Time course of blood–brain barrier differentiation and interaction with pericytes. B- Relative permeability of endothelial monolayers to lucifer yellow after 16 days of culture with different cell types. The monolayers were first cultured with glial cells for 12 days and then cultured for 4 days (i) alone ("soloculture"), (ii) with glial cells (" + glial cells"), (iii) with bovine pericytes (" + bovine pericytes") and (iv) with porcine NN pericytes (" + NN pericytes"). The results represent the mean ± s.e.m of 3 independent experiments. For statistical purposes, a one-way analysis of variance was followed by a Tukey test (*p* < 0.05) with n = 3. The reference permeability coefficient value was 0.51 × 10^-3^ cm/min, corresponding to the coculture condition (+ glial cells). N.S.: non-significant.

In order to investigate the effect of pericytes on BBB permeability, endothelial cells were cocultivated with pericytes. Lucifer yellow is routinely used as a paracellular marker for checking the tightness of junctions between endothelial cells. The BBB's permeability was assessed by using this fluorescent marker following a two-step process (Figure 3A).

An initial 12-day period of endothelial cell-glial cell coculture enabled BBB differentiation. During this first phase, endothelial cells come to express most of the BBB's features; they form a monolayer of non-overlapping, contact-inhibited cells expressing tight junction proteins, monoamine oxidase and γ-glutamyl transpeptidase. These filter-supported monolayers exhibited high transendothelial, electrical resistance and were impermeable to small hydrophilic molecules such as sucrose, inulin and LY [14,22,23].

To study the effect of pericytes on BBB permeability, differentiated endothelial cells were cultivated for an additional four days with pericytes from the different porcine genotypes. This coculture was timed to enable the endothelial cells and pericytes to interact *via* soluble factors. As controls, a number of filters were maintained on glial cells for an additional four days and others were transferred onto "empty" wells (a condition referred to as "soloculture"). The pericytes' growth rates under these conditions were identical, since the density of pericytes extracted from all three porcine genotypes was similar after four days of coculture with endothelial cells (~600,000 pericytes/well). The endothelial monolayers' permeability was therefore assessed after 16 days of culture (Figure 3A).

Control monolayers cocultured for 16 days with glial cells showed low permeability to LY (permeability coefficient (P_e_) = 0.51 × 10^-3^ cm/min, Figure 3B). In contrast, monolayers which had been cultivated alone for 4 days (after 12 days of differentiation with glial cells) showed two-fold greater LY permeability (Figure 3B), confirming that glial cells are required to maintain the BBB's characteristically low permeability [24,25].

To avoid species-related variations in the results, differentiated bovine endothelial cells were cultivated with pericytes of wild-type bovine and porcine origins for 4 days. Although the *in vitro* BBB model contains bovine endothelial cells, the permeability values obtained with wild-type porcine and bovine pericytes were similar (Figure 3B). This finding demonstrated that the pericytes' origin did not influence the permeability of the bovine endothelial cell layer. By ruling out species-related variation, this experiment enabled us to subsequently test the three types of porcine pericytes with confidence.

**Figure 4:**
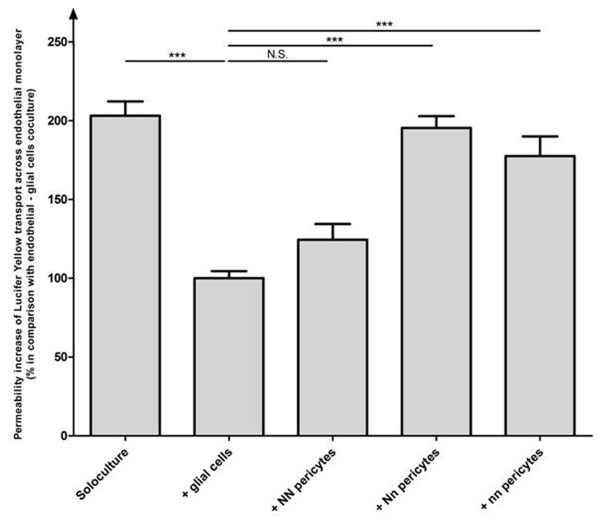
***In vitro*blood–brain barrier permeability investigation.** The relative permeability of endothelial monolayers to Lucifer Yellow was assessed after 16 days of culture with different cell types. The monolayers were first cultured with glial cells for 12 days and then cultured for 4 days (i) alone ("soloculture"), (ii) with glial cells (" + glial cells"), and (iii) with porcine pericytes from the different genotypes (" + NN pericytes", " + Nn pericytes" and + "nn pericytes"), according to the protocol described in Figure 3A. The results represent the mean ± s.e.m of 3 independent experiments. For statistical purposes, a one-way analysis of variance was followed by a Tukey test (*p* < 0.05) with n = 6. The reference permeability coefficient value was 0.28 × 10^-3^ cm/min, corresponding to the coculture condition (+ glial cells). N.S.: non-significant.

The P_e_ for pericytes from NN pigs was similar to that for coculture with glial cells (0.51 × 10^-3^ and 0.28 × 10^-3^ cm/min, respectively; Figures 3B and 4). This P_e_ was considered to be the reference value (i.e. 100%). These data showed that pericytes from wild-type animals are able to maintain the low BBB permeability of endothelial monolayers after 4 days of culture in the absence of glial cells.

When endothelial cells were cultivated with pericytes from mutated pigs, the Pe values were markedly higher (Figure 4). Pericytes from both types of homozygous (nn) and heterozygous (Nn) mutant pigs gave rise to a similar increase in permeability (with a 1.8-fold and a 1.9-fold increase over the reference value, respectively; Figure 4). Hence, unlike pericytes from wild-type pigs, cells extracted from mutated animals were associated with enhanced BBB permeability *in vitro*.

#### Determination of vascular endothelial growth factor levels in the culture supernatants

Although the two cell types are not in direct contact, the experimental design allows crosstalk between them *via* soluble factors. Therefore we next sought to establish whether a soluble factor was responsible for the effect of mutant pericytes on endothelial cell permeability; vascular endothelial growth factor (VEGF) is a candidate for this crosstalk [26]. Indeed, enhancement of permeability can be due to an opening of the tight junctions or to an increase in non-specific vesicular transport. The increase in vesicle formation observed in brain endothelial cells in mutated pigs, as demonstrated by the electron microscopy study (Figure 1D), could be due to an increase in VEGF secretion by pericytes. Esser *et al.*[27] demonstrated that VEGF can increase endothelial permeability by inducing the formation of fenestrations but also by stimulating vesicle formation in endothelial cells, as observed here in brain endothelium in mutated pigs. To test this hypothesis, the coculture media were collected and assayed (radio receptor assay) for secreted VEGF.

The mean ± SD VEGF concentration was 1567 ± 88.2 pg.mL^-1^ in NN pericyte wells (the abluminal compartment) and 1733 ± 60.1 pg.mL^-1^ in Nn pericyte wells. In contrast, a markedly lower VEGF concentration was found in nn pericyte wells (500 ± 57.74 pg.mL^-1^). These results did not correlate with the above-described BBB permeability data (Figure 4) and make it unlikely that VEGF-A was responsible for the permeability increase seen with pericytes from mutant pigs.

## Discussion

A study in stress-susceptible pigs revealed particularly evident changes in skeletal muscle vessels [2]. In the present work, similar changes in brain microvessel endothelium were observed, suggesting that both cerebral vessels and skeletal muscle components have abnormalities. Most of the changes involved the parenchyma surrounding the vessels, with enlarged perivascular spaces, oedema and the accumulation of lipids, in association with swollen astrocytic endfeet. These features probably testify to vascular dysfunction, since no changes were observed in the remote parenchyma. These morphological data in stress-susceptible pigs suggest that modifications in NVU function are involved in PSS.

The BBB plays a crucial role in the maintenance of brain homeostasis by preventing non-specific transport of blood-borne molecules. Indeed, leakage of serum components into the brain parenchyma can lead to progressive vasogenic oedema formation [28]. This barrier function is performed by the capillary endothelial cells, the cellular environment of which is essential in establishing and maintaining their phenotype. Pericytes form part of this environment. Indeed, pericytes not only regulate the capillary flow [29] (as do their smooth muscle cell counterparts) but are also involved - along with glial cells - in inducing BBB differentiation and regulating endothelial cell function [30-32]. Our ultrastructural results in *ryr1*-mutated pigs demonstrate important modifications of astrocytes at the level of the BBB, as shown by astrocytic endfeet swelling; on the other hand, pericytes associated with the BBB did not exhibit remarkable changes. This observation does not correlate with mural cell modifications observed in peripheral vessels [2]. The aim was therefore to check the proper function of pericytes in maintaining BBB features, and in particular their ability to maintain BBB permeability *in vitro*.

Since the cellular mechanisms of cell-cell communication are easier to study with cell culture techniques, pericytes from the three genotypes of stress-susceptible and non-stress susceptible pigs were tested for their influence on BBB permeability *in vitro*. This experimental configuration enabled us to addressing the question of potential functional changes at the BBB in the presence of the pericytes.

*In vitro*, the three types of porcine pericytes had the same morphological characteristics as bovine pericytes and all were positive for NG2 and alpha-smooth muscle actin. The observed morphological and immunochemical features, the high gamma-glutamyl transpeptidase activity (in contrast to smooth muscle cells), the isolation protocol (which mostly selects the vascular tree's capillary fractions) and use before the third passage (to avoid trans- or de-differentiation) enables us to state with confidence that the cells isolated here were indeed brain pericytes and not smooth muscle cells [21]. All three types of pericytes had the same cell density on the day of the experiment. *In vitro*, all types of pericytes behaved in an equivalent manner, regardless of the genotype of the animal from which they were isolated; this indicated that pericytes were not modified in their morphology or growth.

To study the pericytes’ effect on BBB permeability, the endothelial monolayers were first cultured for 12 days with glial cells [14,22]. This step enables the BBB-specific differentiation of endothelial cells so that the latter (i) form a regular monolayer of contact-inhibited cells, (ii) express tight junction proteins and (iii) are impermeable to small hydrophilic molecules.

After this initial phase, endothelial cells were transferred onto wells containing brain pericytes, with which they were able to interact (*via* soluble factors) for 4 days. This period is sufficient to enable pericytes and endothelial cells to influence each other: preliminary work in our laboratory using the same protocol has shown that pericytes are able to modulate the expression of BBB transporters [12]. In this case, *MRP6* mRNA expression in endothelial cells appeared to be re-induced by the presence of pericytes for 2, 4 and 6 days, indicating that pericyte-secreted soluble factors were able to trigger *MRP6* transcription in brain capillary endothelial cells. Moreover, pericytes can reinforce the low BBB permeability to an integrity marker in a three-cell culture model, in synergy with glial cells *in vitro*[10,21].

Endothelial cells exhibiting BBB features present low permeability to LY, a probe of monolayer integrity *in vitro*. The endothelial cells' LY permeability was low after culture for 4 days with glial cells but was dramatically higher in the absence of glial cells. These data confirm that glial cells are required for maintaining the BBB's characteristics, as previously described [24,25].

When cultured for 4 days with pericytes from wild-type cows and pigs, the monolayer still presented low LY permeability (similar to that of the monolayer maintained with glial cells). These data show that pericytes are able to maintain (at least for 4 days) the BBB's low permeability to LY. Indeed, pericytes are able to secrete factors which may be involved in BBB differentiation and maintenance, such as TGF-β [13] and angiopoietin-1 [33].

Since pericytes from wild-type (NN) animals were associated with essentially the same BBB permeability as bovine pericytes in a syngenic coculture, we were able to test the different genotypes of porcine pericytes on BBB endothelial cells in our model. In contrast, the pericytes from *ryr1* mutated pigs were responsible for an increase in BBB permeability. On the day of permeability assessment, the pericyte density was equivalent for the three different genotypes, so the observed permeability changes cannot be attributed to cell density differences in the wells. These findings led us to infer that the BBB is likely to be more permeable to blood-borne molecules in stress-susceptible pigs. This phenomenon could be part of the process leading to the enlargement of perivascular spaces. Impaired cell-cell communication within the NVU can lead to brain modifications [32].

The greater LY permeability observed with pericytes from mutant pigs could be related to their inability to maintain the BBB's characteristics and/or their ability to secrete soluble factors. Vascular endothelial growth factor (VEGF) is a potential candidate because it is well-known potent vascular permeabilizing factor, which can induce an increased vesicle formation in endothelial cells [27]. To verify the latter hypothesis, we assayed for VEGF-A in the culture media taken from the various conditions. The potent endothelial permeabilizing factor VEGF is known to be secreted by pericytes [34]. Our results indicated that the greater LY permeability observed with pericytes from mutant pigs could not be accounted for by VEGF-A secretion, since levels of this factor were higher in the NN control condition than in mutant pericyte-endothelial cell cocultures. In order to draw firm conclusions on this matter, differential secretome analyses are currently under investigation in the lab, using conditioned media; such an approach could identify candidate soluble factors and investigate their secretion by the different types of pericytes.

Our results demonstrate that brain pericytes from *ryr1* mutated animals have functional defects because they are not able to maintain BBB permeability as do pericytes from healthy pigs, but this observation cannot be directly linked with *ryr1* mutation because they seem not to express this gene, suggesting a side-effect of *ryr1* mutation instead. Impaired calcium signalling in the brain can be responsible for BBB modifications [35]: indeed, impaired calcium signalling in astrocytes can for example lead to glutamate release and pericytes are sensitive to glutamate [29]. Multiple ways could lead to pericyte dysfunction in brain capillaries in the context of impaired calcium signalling, influencing BBB maintenance in PSS, but this could be completely silent.

## Conclusions

Our results demonstrate the brain pericytes from *ryr1* mutated pigs - even if they seem not affected by this condition at the ultrastructural level - are not able to maintain BBB integrity *in vitro* in comparison with pericytes from wild-type animals. It can therefore be inferred that BBB permeability is increased in stress-susceptible pigs, so that it becomes more permeable to blood-borne molecules, and this phenomenon can be part of the process leading to enlarged perivascular spaces in PSS condition. Pericytes are now emerging as crucial cellular elements of the BBB, interacting with the other components of the NVU for maintaining proper cerebral function, and our results show that their involvement should not be neglected in the development of BBB changes in pathological conditions. In this frame, pericytes could be considered as new cellular targets to be modulated in pathological states, but further studies are necessary for unravelling the pathways leading to NVU and BBB changes.

## Abbreviations

BBB: Blood–brain barrier; GFAP: Glial fibrillary acidic protein; MH: Malignant hyperthermia; LY: Lucifer yellow; PSS: Porcine stress syndrome; NG2: Nerve-glial antigen 2; NVU: Neurovascular unit; PVS: Perivascular space; VEGF: Vascular endothelial growth factor; vWF: von Willebrand Factor; TGF-β: Transforming growth factor beta; MRP6: Multidrug resistance protein-6.

## Misc

This work is dedicated to the memory of Jean Plouët (deceased May 30, 2008)

## Competing interests

The authors declare that they have no competing interests.

## Authors’ contributions

Generated the original hypothesis: MMR. Provided brain samples and discussed the project: MF. Performed and analyzed experiments: EV, LD, CG, JP, FG. Wrote the paper: EV MPD RC MC. All authors read and approved the final manuscript.
